# Supplementary material for: Higher social tolerance is associated with more complex facial behavior in macaques
Source: eLife. 2023 Oct 3;12:RP87008. doi: 10.7554/eLife.87008 (PMC10547472; doi:10.7554/eLife.87008)
Supplement: Supplementary file 1. [file elife-87008-supp1.docx]

Supplementary file 1

Supplementary Information for

**Higher social tolerance is associated with more complex facial behavior in macaques**

Alan V. Rincon, Bridget M. Waller, Julie Duboscq, Alexander Mielke, Claire Pérez, Peter R. Clark, Jérôme Micheletta

Alan V. Rincon
Email: [avrincon1@gmail.com](mailto:avrincon1@gmail.com)

Jérôme Micheletta
Email: [jerome.micheletta@port.ac.uk](mailto:jerome.micheletta@port.ac.uk)

## Action Unit list

**Table 1:** Action Units (AU) and Descriptors (AD) observed and coded in the study.

| Action Unit/Descriptor | Description |
| --- | --- |
| AU1+2 | Brow raiser |
| AU41 | Glabella (brow) lowerer |
| AU5 | Upper lid raiser |
| AU6 | Cheek raiser |
| AU8 | Lips toward each other |
| AU9 | Nose wrinkler |
| AU10 | Upper lip raiser |
| AU12 | Lip corner puller |
| AU16 | Lower lip depressor |
| AU17 | Chin raiser |
| AU18^*^ | Lip pucker |
| AU25 | Lips parted |
| AU26 | Jaw drop |
| AU27 | Jaw stretch |
| AU43 | Eyelid droop |
| EAU1 | Ears forward |
| EAU2 | Ears elevator |
| EAU3 | Ears flattener |
| AD19 | Tongue show |
| AD29 | Jaw thrust |
| AD59 | Head toss |
| AD101 | Scalp retraction |
| AD181^†^ | Lipsmack |
| AD182^†^ | Teeth chatter |
| AD184^†^ | Jaw wobble |
| AD185^‡^ | Jaw oscillation |
| ^*^AU18i and AU18ii were combined because it was difficult to reliably distinguish between them when coding videos.^†^Excluded in favor of AD185, which allows for a more detailed description of facial behavior when combined with other AUs.^‡^New AD not previously described in MaqFACS. Denotes stereotyped up-and-down movement of the jaw. | |
